# Determination of optimal gamma radiation dose for mutation breeding in ’Sabz’ fig (*Ficus carica* L.) cuttings based on radiosensitivity and phenotypic changes

**DOI:** 10.1371/journal.pone.0313017

**Published:** 2025-01-15

**Authors:** Ali Akbar Ghasemi-Soloklui, Mojtaba Kordrostami, Moslem Jafari

**Affiliations:** 1 Nuclear Agriculture Research School, Nuclear Science and Technology Research Institute (NSTRI), Karaj, Iran; 2 Fig Research Station, Fars Agricultural and Natural Resources Research and Education Center, Agricultural Research, Education and Extension Organization (AREEO), Estahban, Iran; China Academy of Traditional Chinese Medicine: China Academy of Chinese Medical Sciences, CHINA

## Abstract

The dried fig cv. Sabz of Iran, distinguishes out among the several fig cultivars for its unique characteristics and excellent properties. The aims to this study were 1) Carefully monitoring the resulting phenotypic changes in growth patterns, leaf morphology, shoot traits, root characteristics, and other relevant traits after irradiated with different gamma rays; 2) Investigating the LD_25_, _50_, _75_ and GR_25_, _50_, _75_ values at different gamma radiation doses for chose optimum dose. According to our results, the LD_50_ was 70 Gy, while the LD_25_ and LD_75_ were approximately 48 and 95 Gy, respectively. Data analysis revealed that higher doses, ranging from 50 to 90 Gy, led to a reduction in leaf area for fig hardwood cuttings compared to those exposed to lower doses of gamma irradiation (10, 20, 30, and 40 Gy). In fig cuttings, the plant height gradually decreased in line with increasing irradiation doses up to 60 Gy. Among the root traits, root number was particularly influenced by higher radiation doses. On other hand, when fig cuttings were exposed to a 40 Gy radiation dosage, the average root count dropped by 50%. However, when fig cuttings were subjected to a 90 Gy radiation dose, the average root count surged by 90.7% in comparison to the control treatment. Additionally, the GR_50_ values were 63 Gy for internode length, 67 Gy for leaf area and 56 Gy for plant height and aerial biomass. However, the GR_50_ values for root number, root volume, and root biomass were 46 Gy, 57 Gy, and 51 Gy, respectively. An analysis based on the GR_25_, GR_50_, and GR_75_ values indicated that plant height, aerial biomass and root biomass exhibited greater sensitivity to radioactivity in comparison to other plant portions of the fig. According to the biological responses in the ‘Sabz’ fig, 60 Gy of gamma radiation is a suitable dose for initial mutagenesis studies.

## Introduction

The ‘Sabz’ fig (*Ficus carica* L. cv. Sabz) is one of Iran’s most valued dried fig cultivars, renowned for its unique characteristics and exceptional qualities. Distinguished by its special green color when fresh, the ‘Sabz’ fig maintains its characteristic attractiveness even after the drying process, which extends its shelf life and concentrates its natural sugars, resulting in a rich, sweet flavor and a pleasant chewy texture [1]. These attributes make it highly desirable in both local and international markets, contributing significantly to the agricultural economy [2, 3]. Despite its commercial success, there is a continuous need to enhance the fruit trees to meet the challenges posed by evolving consumer preferences, climate change, and emerging pests and diseases [4, 5]. Traditional breeding methods in figs are limited due to the plant’s long juvenile period, complex genetics, and parthenocarpy nature, which complicates sexual reproduction and seed-based propagation [6, 7]. Therefore, alternative approaches like mutation breeding are essential to introduce genetic variability and develop new cultivars with improved traits such as disease resistance, enhanced fruit quality, and better adaptability to environmental stresses [8, 9].

Gamma radiation is a widely used mutagenic agent in plant breeding programs due to its ability to induce mutations by causing changes in the DNA sequence [10]. As a form of ionizing radiation, gamma rays have high penetration power [11], enabling them to reach deep into plant tissues and affect a large number of cells, including those in the meristematic regions [8]. This increases the likelihood of inducing beneficial mutations that can be passed on to future generations, facilitating the development of novel clones with unique and desirable characteristics [9]. The application of gamma radiation in mutation breeding has several advantages. It allows for the creation of genetic variability without altering the overall genetic makeup of the cultivar, preserving desirable traits while introducing new ones [12]. Moreover, gamma radiation can be applied to vegetative parts such as cuttings, which is particularly useful for vegetatively propagated crops like figs [13]. This method accelerates the breeding process compared to traditional methods, which are time-consuming and less efficient in inducing genetic changes.

However, the successful use of gamma radiation requires careful optimization of the dosage, as excessive doses can lead to severe damage or death of plant tissues, while insufficient doses may not induce significant mutations [14]. Determining the optimal radiation dose is crucial to balance mutation induction with plant survival and growth. Key parameters such as the lethal dose (LD) and growth reduction (GR) values are essential for assessing the radiosensitivity of plant materials. The LD values (LD_25_, LD_50_, LD_75_) represent the doses that cause 25%, 50%, and 75% mortality, respectively, while the GR values (GR_25_, GR_50_, GR_75_) indicate the doses that result in corresponding percentages of growth reduction [15, 16]. These parameters provide valuable insights into the plant’s tolerance to radiation and help in selecting appropriate doses for mutation breeding. Previous studies have utilized gamma radiation in fig breeding programs to induce mutations resulting in improved traits. For instance, Flaishman et al. [7] reported on the development of a new fig variety with altered fruit maturation characteristics through gamma irradiation. Özen et al. [17] observed changes in fruit weight traits in figs exposed to gamma radiation, and Aksoy [2] highlighted the potential of gamma radiation to induce dwarfism and accelerate fruiting in figs. Despite these advancements, the radiosensitivity and optimal gamma radiation doses for different fig cultivars, including ‘Sabz’, remain underexplored [18, 19].

Understanding the specific responses of ‘Sabz’ fig cuttings to gamma radiation is essential for the successful application of mutation breeding in this cultivar. Investigating the effects of different gamma radiation doses on survival rates, growth patterns, leaf morphology, shoot traits, root characteristics, and other relevant traits will provide valuable data for optimizing mutagenesis protocols. Therefore, the objectives of this study were: a) to carefully monitor the resulting phenotypic changes in ‘Sabz’ fig cuttings after irradiation with different gamma radiation doses, focusing on growth patterns, leaf morphology, shoot traits, root characteristics, and other relevant traits. This will help identify the morphological and physiological effects of gamma radiation on the plant and determine how different doses influence these traits; b) to investigate the LD_25_, LD_50_, LD_75_, and GR_25_, GR_50_, GR_75_ values at different gamma radiation doses to select the optimal dose for mutation induction in ‘Sabz’ fig cuttings. Determining these values will provide insights into the radiosensitivity of the cultivar and help establish a dose-response relationship, which is critical for effective mutation breeding. By achieving these objectives, this research aimed to contribute to the advancement of fig breeding programs by providing essential data on the radiosensitivity of ‘Sabz’ fig and establishing a foundation for the development of improved cultivars with enhanced traits through gamma radiation-induced mutagenesis. The findings will not only benefit the ‘Sabz’ cultivar but also provide a framework that can be applied to other fig cultivars and similar vegetatively propagated crops.

## Material and methods

### Plant material

The ‘Sabz’ fig (*Ficus carica* L.) was selected for this study. On January 1, 2022, uniform-sized one-year-old cuttings of the ‘Sabz’ fig were collected from thirty-year-old trees at the Estahban Fig Research Station in the southern province of Fars, Iran. The Estahban Fig Research Station is a government-affiliated research facility, and access to the site for scientific purposes does not require special permits beyond the standard institutional approval. No additional permits were required for this work, as the research was conducted within the station’s designated experimental plots. All procedures adhered to local and national regulations governing agricultural research.

To ensure sterility, the samples were immersed in a solution of 4000 ppm benomyl (Ariashimi Co., Zahedan, Iran) for 5 minutes. Subsequently, the cuttings were wrapped in moist paper, packed in plastic bags, and stored at 5°C for transportation from the field to the laboratory.

### Gamma irradiation

For the gamma irradiation experiment, eleven groups of fig cuttings were prepared, including the control group and ten treatment groups (10, 20, 30, 40, 50, 60, 70, 80, 90, and 100 Gy). Each treatment consisted of 150 cuttings, with a total of 1,650 cuttings used in the experiment. The experiment was arranged in a completely randomized design (CRD) with three replicates per treatment, each replicate consisting of 50 cuttings. Following exposure to gamma radiation, the basal ends of the cuttings were treated with indole-3-butyric acid (3000 ppm in 50% ethanol) and planted in plastic bags containing untreated cuttings as a control that had already been planted in sand. All cultured cuttings from each treatment were kept in a controlled greenhouse with a temperature of 20 to 25°C and a relative humidity of 60 percent until rooting of the cuttings commenced.

### Measurements of plant growth traits

Measurements of plant height (cm), internode length (cm), number of nodes, and leaf width (mm) were taken four months after planting under optimal conditions. Plant height was measured from the base of the stem to the tip of the tallest shoot using a measuring tape. Internode length was measured between two consecutive nodes using a ruler. The number of nodes was counted manually for each plant. Leaf width was measured at the widest point using a digital caliper.

The leaf area (mm^2^) was determined using ImageJ software ver. 1.53 and cross-verified with an automatic electronic leaf area meter (model LI-3000, LI-COR Biosciences, Lincoln, NE, USA). Root properties such as the number of roots were counted manually after carefully washing and separating them from the cutting base. The highest root length (cm) was measured using a ruler. Root volume (cm^3^) was calculated using the water displacement method with a graduated cylinder. The fresh weight (g) of the roots was assessed using a digital balance with an accuracy of 0.01 g. To determine the dry weight of the aerial biomass and root biomass, the samples were dried in an oven for 72 hours at 65°C and then weighed using a Mettler Viper BC analytical scale (Mettler-Toledo, Columbus, OH, USA). The survival percentage was calculated by dividing the number of dead cuttings by the total number of treated cuttings for each gamma irradiation dose. The mean survival percentage, as well as the lethal dose (LD_25_, LD_50_, and LD_75_), and growth reduction (GR_25_, GR_50_, and GR_75_) for shoot, root, and leaf were estimated using the Ghasemi-Soloklui [20] method.

### Statistical analysis

Data were analyzed using one-way analysis of variance (ANOVA) with the statistical software SAS version 9.4. LSD test was employed to determine the significance of differences between the mean values at the 1% level of significance.

## Results

### Effect of mutagenesis on survival percentage of hardwood cutting

The survival percentage of hardwood cuttings decreased progressively with increasing doses of gamma irradiation (Table 1). The control group and the 10 Gy treatment both exhibited the highest survival percentage (98.8%), indicating that low-dose gamma radiation had negligible effects on cutting viability. However, survival rates began to decline significantly at doses above 40 Gy. At 50 Gy and 60 Gy, survival percentages dropped to 81.84% and 80.6%, respectively, suggesting that moderate doses start to have a detrimental impact on cutting viability.

**Table 1:** Effect of gamma irradiation on survival of hardwood cuttings of *Ficus carica* L. cv. Sabz.

Dose (Gy)	Survival percentage	Percent survival over control (%)	Percent reduction over control (%)
0	98.8	100	-
10	98.8	100	0
20	97.5	98.68	1.31
30	98.8	100	0
40	96.25	97.43	2.56
50	81.84	82.84	17.15
60	80.6	81.57	18.42
70	81.25	82.57	17.76
80	56.29	56.9	43.02
90	19.25	19.48	80.51
100	0	0	100

The most drastic reductions were observed at higher doses: 80 Gy resulted in a survival percentage of 56.29%, and 90 Gy led to a mere 19.25% survival rate. The 100 Gy treatment resulted in complete mortality (0% survival). This trend indicates that higher doses of gamma radiation cause substantial cellular and DNA damage, leading to increased mortality. These findings have important implications for mutation breeding programs, as they highlight the need to balance the induction of mutations with the viability of the plant material.

### Determination of LD_25_, LD_50,_ and LD_75_ of mutagens

**Fig 1:**
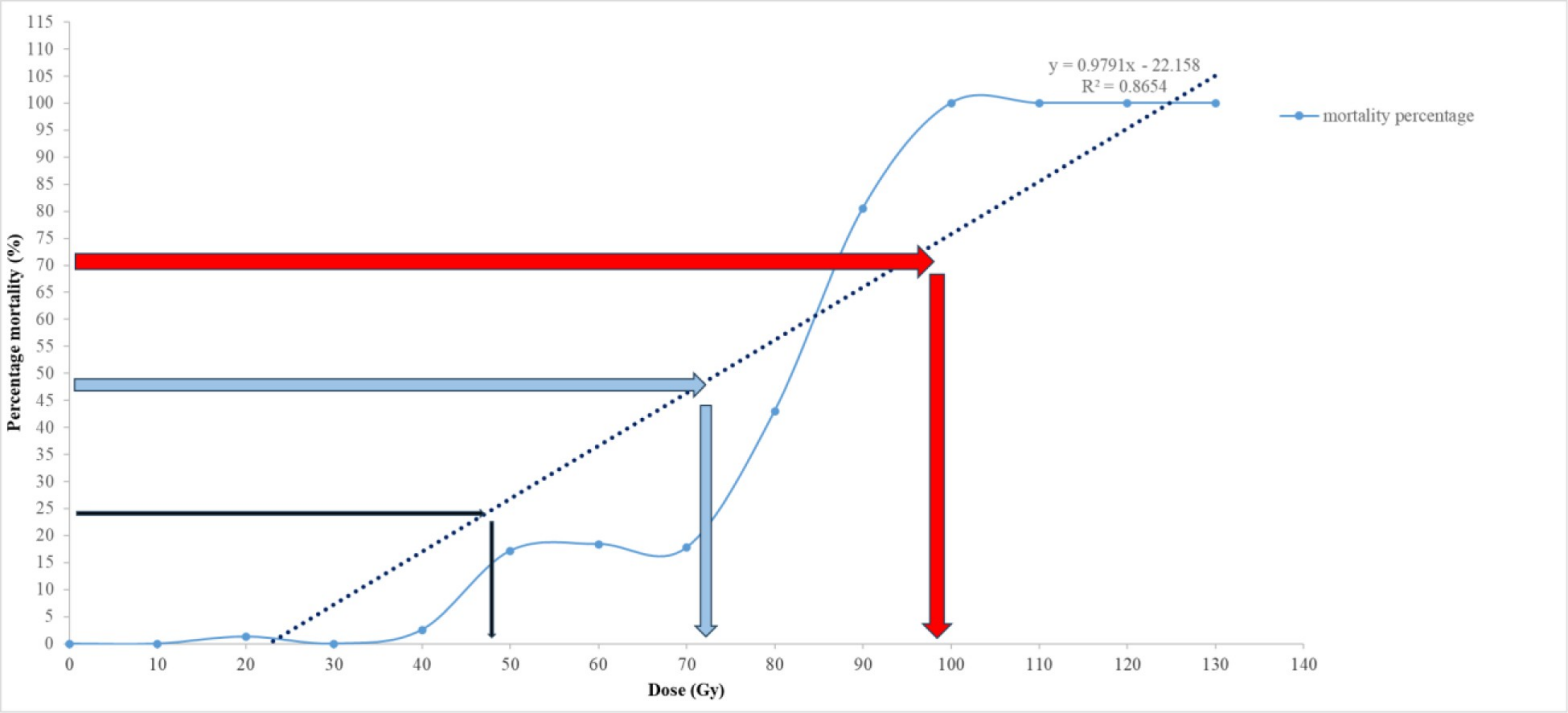
Analysis Lethal dose *Ficus carica* L. cv. Sabz irradiated with different doses of gamma-ray. The regression equation obtained was Y = 0.9791x - 22.158, where Y represents the survival percentage, and x represents the irradiation dose in Gy. The coefficient of determination (R^2^) was 0.86, indicating a strong correlation between radiation dose and survival percentage.

The lethal doses (LD_25_, LD_50_, and LD_75_) were determined using probit analysis and linear regression modeling of the survival percentage data against the gamma irradiation doses (Fig 1).

The LD_50_ value, representing the dose at which 50% of the cuttings are expected to die, was calculated by setting Y = 50% in the regression equation and solving for x, resulting in an LD_50_ of approximately 70 Gy. Similarly, the LD_25_ and LD_75_ values, indicating 25% and 75% mortality rates, were calculated by setting Y = 75% and Y = 25%, yielding doses of approximately 48 Gy and 95 Gy, respectively.

The rationale for using this regression model is based on its ability to accurately represent the dose-response relationship and predict lethal doses for the population. These LD values are crucial for determining appropriate radiation doses for mutagenesis studies, balancing mutation induction with plant survival.

### Effects of mutagens on leaf traits

**Fig 2:**
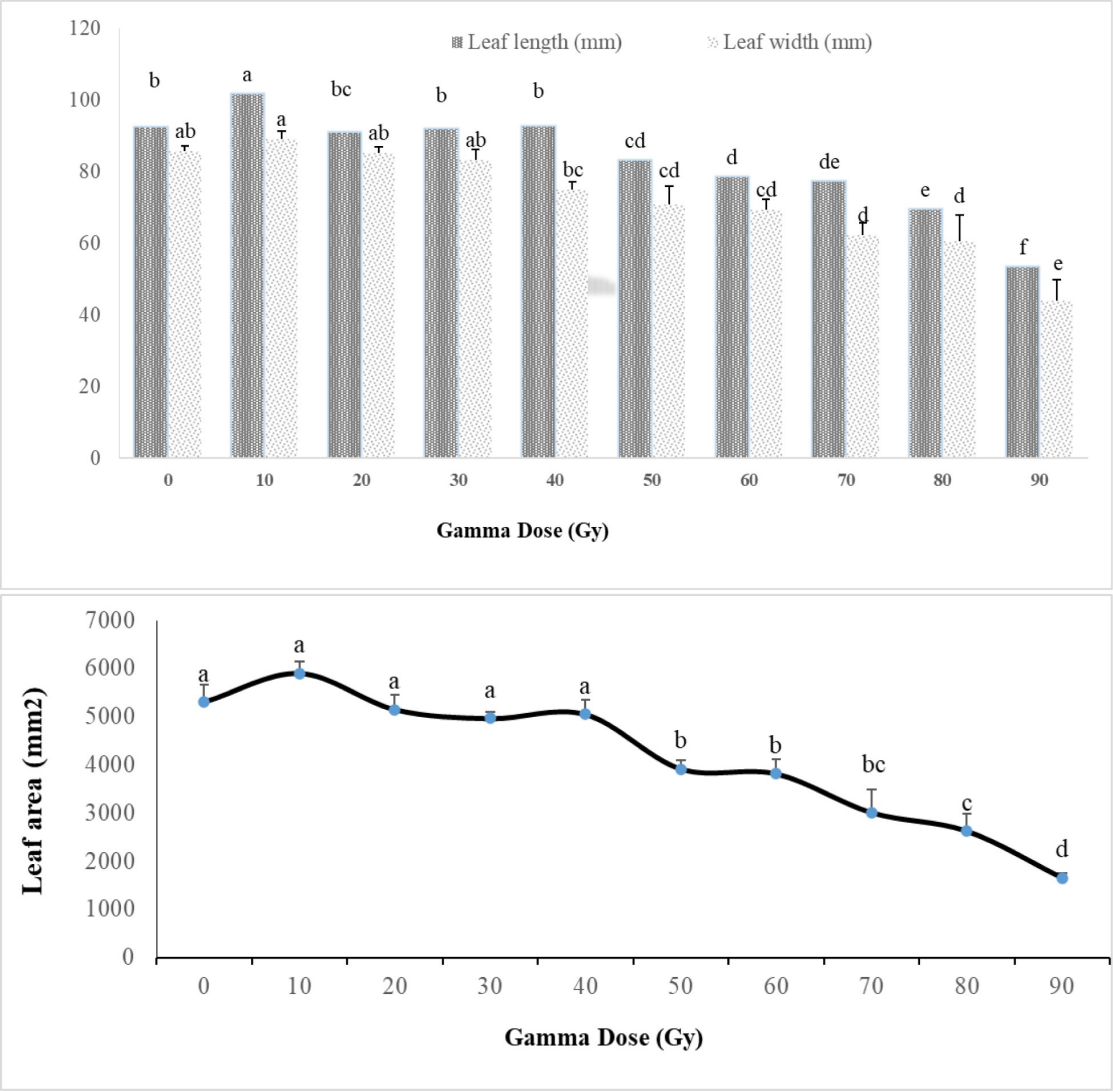
Effects of different gamma irradiation doses on leaf traits in *Ficus carica* L. cv. Sabz. Data analysis revealed that higher doses, ranging from 50 to 90 Gy, led to a reduction in leaf area for fig hardwood cuttings compared to those exposed to lower doses of gamma irradiation (10, 20, 30, and 40 Gy). Our results indicate that the smallest leaf area was observed in response to higher doses of gamma radiation, particularly at 80 Gy (2623 mm^2^), 90 Gy (1652 mm^2^), and other lower doses (5306 mm^2^). Moreover, gamma ray treatments such as 50, 60, and 70 Gy had a moderate impact on the leaf area of fig cuttings.

Based on the findings presented in Fig 2, it is apparent that various doses of gamma radiation have significant impacts on the physical characteristics of fig leaves, specifically their area, length, and width.

Fig 2 also demonstrates that the average length and width of ‘Sabz’ fig cutting leaves were significantly affected by increasing doses of gamma rays. A gradual decrease in both leaf length and width was observed with higher radiation doses during the experiment. The control treatment and low doses of gamma radiation exhibited the highest leaf length (92 to 101 mm) and leaf width (83 to 89.3 mm), while the lowest leaf length (53 to 83 mm) and width (44 to 75 mm) in fig cuttings were observed within the 50 to 90 Gy range of gamma treatments.

### Effects of mutagens on shoot traits

**Fig 3:**
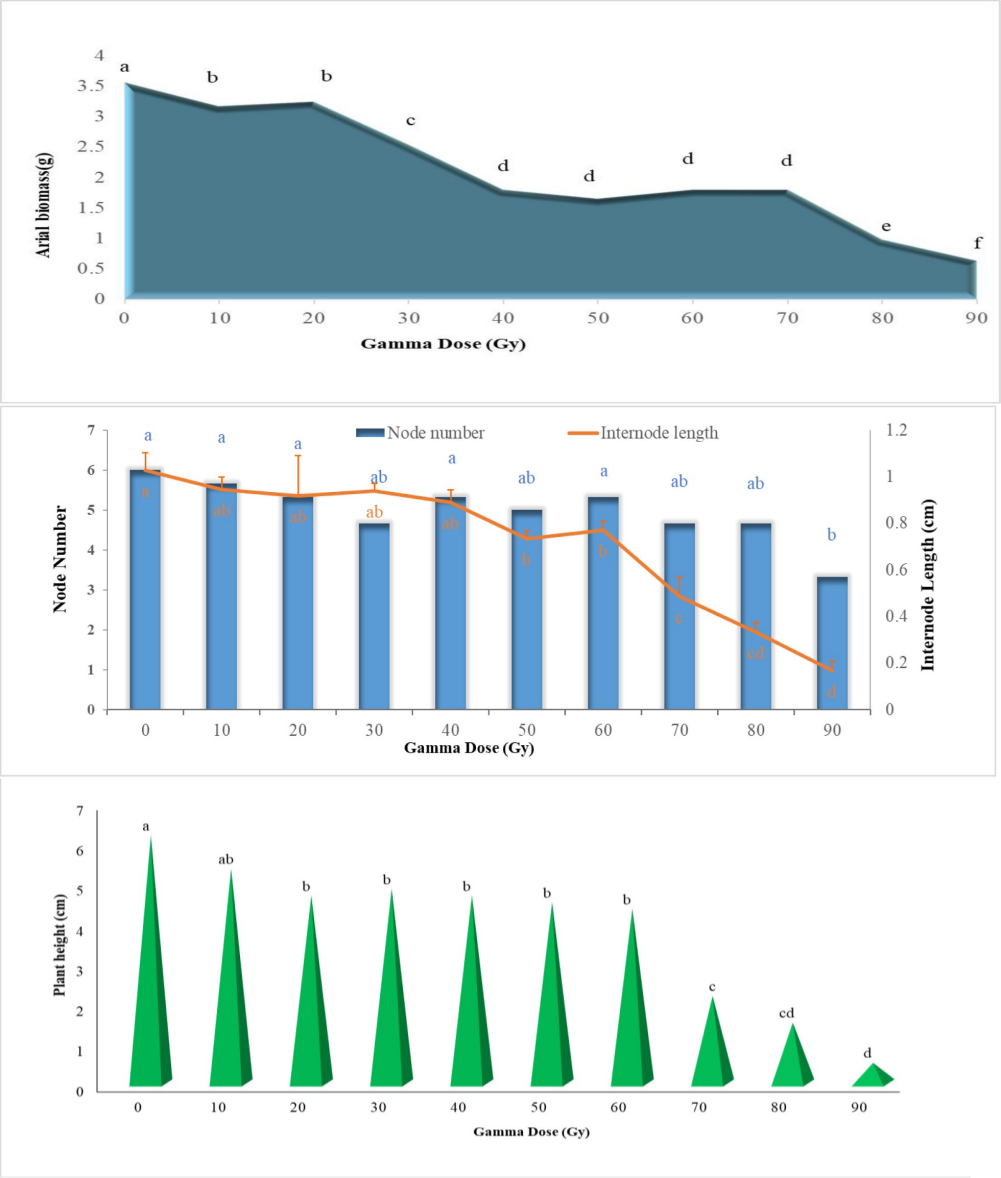
Effects of different gamma irradiation doses on shoot traits in *Ficus carica* L. cv. Sabz.

The effects of different gamma radiation doses on plant height were depicted in Fig 3.

Upon examining Fig 3, it is evident that the gamma ray treatments had a negative effect on plant height. In fig cuttings, the plant height gradually decreased in line with increasing irradiation doses up to 60 Gy. However, a sudden decrease (from 2.16 to 4.33 cm) in shoot length was observed when applying gamma radiation doses from 60 to 70 Gy in the Sabz cultivar. Furthermore, the response of other vegetative growth parameters in hardwood cuttings to varying doses of gamma radiation, such as internode length and internode number, was evaluated and presented in Fig 3. In the case of ‘Sabz’ cuttings, low and moderate gamma doses did not have a significant effect on the number of nodes, but significant decreases were observed when exposed to 90 Gy of radiation. The highest internode length (1.02 cm) was recorded in untreated cuttings (0 Gy), while the lowest internode lengths were recorded at 70 Gy (0.48 cm), 80 Gy (0.33 cm), and 90 Gy (0.16 cm), respectively.

### Effects of mutagens on root traits

According to Table 2, the root characteristics of fig cuttings exposed to various dosages of gamma rays exhibited distinct responses compared to both control and treated hardwood cuttings. As depicted in Table 2, the fresh weight and root biomass of plants subjected to gamma irradiation, along with their root volume, highest root length, and root number, gradually diminished as the gamma doses increased from 10 to 90 Gy. Among the root traits, root number was particularly influenced by higher gamma radiation doses. On other hand, when fig cuttings were exposed to a 40 Gy radiation dose, the average of root count dropped by 50%. However, when fig cuttings were subjected to a 90 Gy radiation dose, the average root count decreased to 90.7% in comparison to the control treatment. The highest root length and fresh weight of the explants improved with an increase in radiation dosage from 0 to 10 Gy. Nevertheless, it’s important to note that only higher doses of gamma radiation negatively impacted the highest root length and root fresh weight of fig cuttings.

**Table 2:** Effects of different doses of gamma irradiation on root characteristics of *Ficus carica* L. cv. Sabz.

Dose (Gy)	Root number	Highest root (cm)	Root volume (cm^3^)	Root biomass (g)
0	32.33±1.6a	25.6±1.85ab	4.00±0.18a	2.40±0.10ab
10	33.00±3.00a	28.00±0.57a	3.16±0.92ab	2.50±0.50a
20	22.66±1.66b	22.31±1.45bc	2.33±0.32bc	1.92±0.25ab
30	22.56±1.16b	23.06±1.03bc	2.50±0.28bc	1.98±0.27ab
40	15.34±2.02c	23.00±1.00bc	2.00±0.28c	1.30±0.04b
50	10.00±1.15c	20.00±1.15c	2.00±0.04c	1.28±0.11bc
60	11.00±3.15c	21.66±1.85bc	2.16±0.16bc	1.36±0.10bc
70	10.65±1.20c	21.33±2.02bc	2.00±0.10c	1.30±0.12bc
80	2.00±0.20d	20.00±1.73c	0.83±0.16d	1.01±0.19bc
90	3.00±0.15d	18.66±0.88c	0.63±0.06d	0.35±0.02c

### Radiosensitive tests

**Fig 4:**
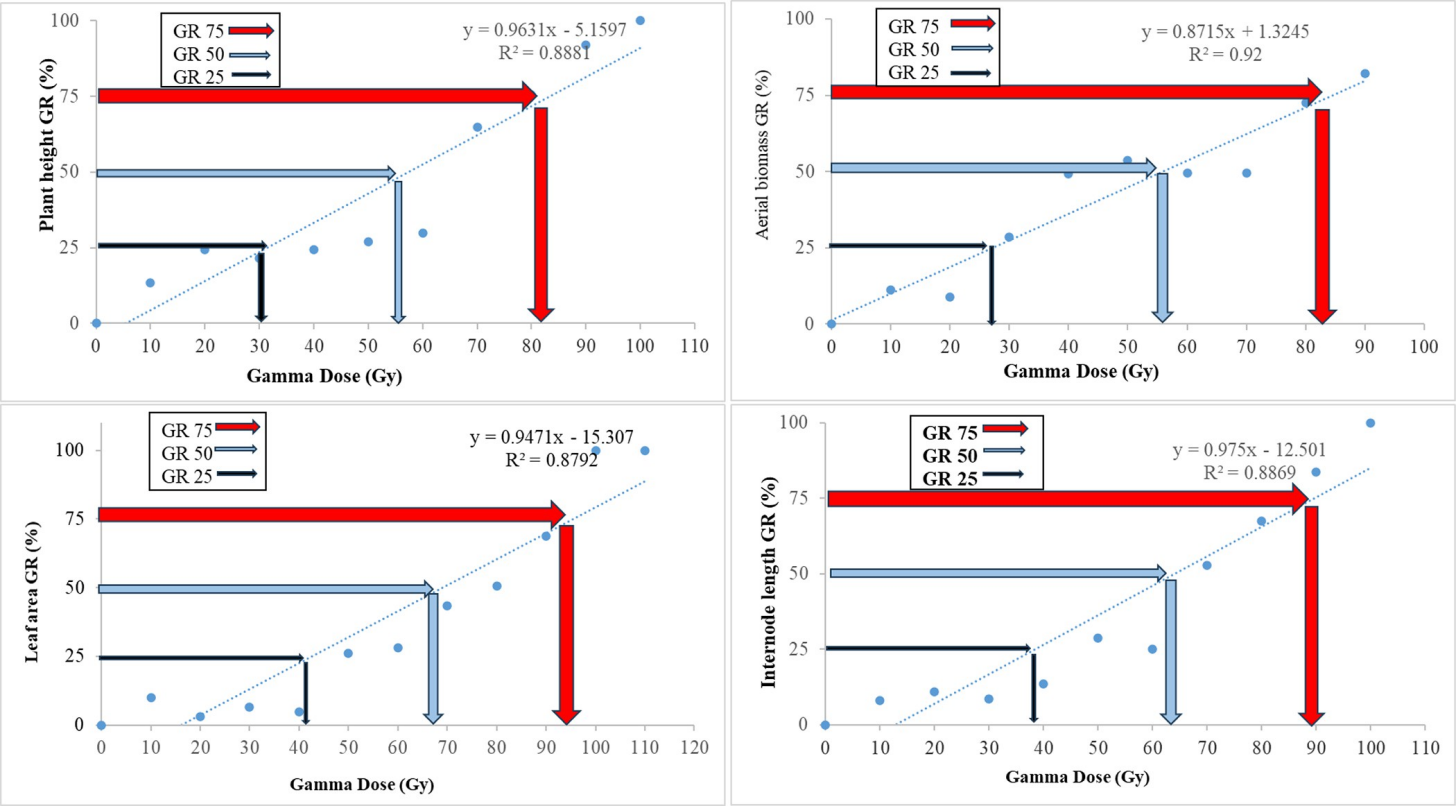
Effect of different gamma ray doses on growth reduction determination of shoot and leaves traits in in hardwood cutting of *Ficus carica* L. cv. Sabz.

**Fig 5:**
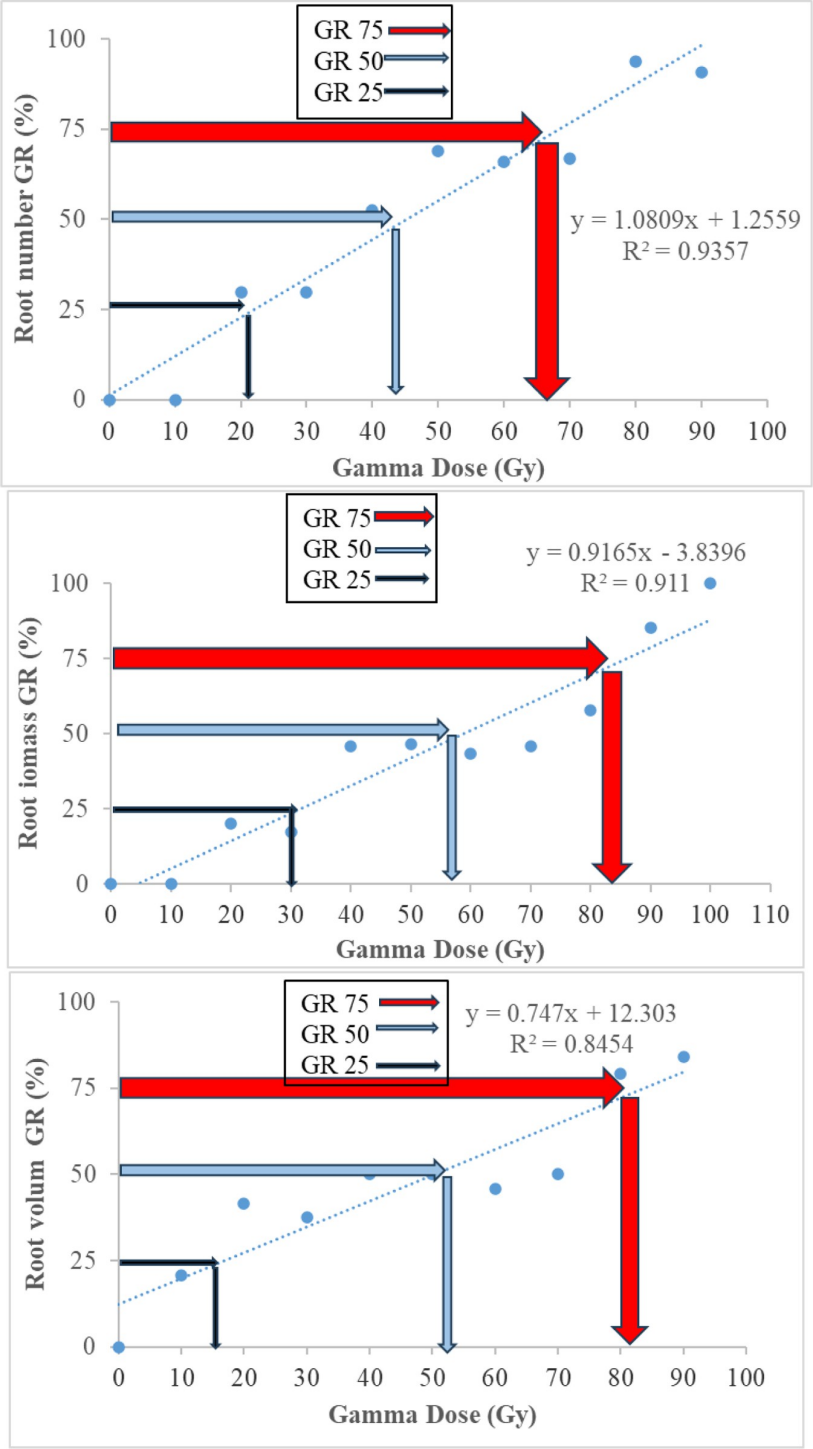
Effect of different gamma ray doses on growth reduction determination of root in hardwood cutting of *Ficus carica* L. cv. Sabz.

**Fig 6:**
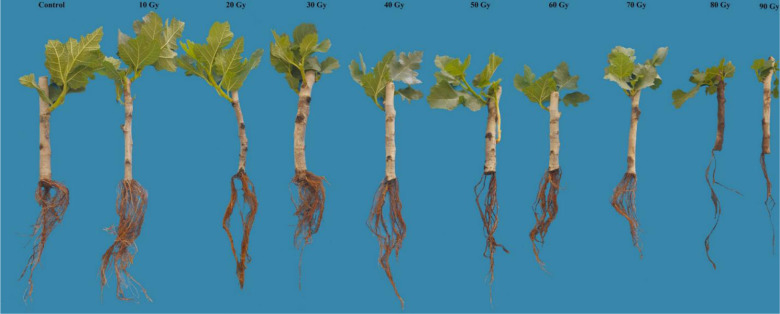
Effects of different gamma irradiation doses on leaf, shoot and root traits in *Ficus carica* L. cv. Sabz.

The growth reduction (GR) values for various plant traits were calculated to assess the sensitivity of ‘Sabz’ fig cuttings to gamma radiation (Figs 4–6).

The GR_25_, GR_50_, and GR_75_ values represented the gamma doses causing 25%, 50%, and 75% reduction in growth, respectively. For aerial traits such as plant height, internode length, leaf area, and aerial biomass, the GR_50_ values ranged from 56 Gy to 67 Gy, indicating moderate sensitivity to gamma radiation. Root traits exhibited GR_50_ values ranging from 46 Gy to 57 Gy, suggesting that root development is more sensitive to radiation than shoot growth. These GR values are significant because they help identify the radiation doses that cause substantial but manageable reductions in growth, which are often associated with higher mutation rates. The selection of an optimal gamma radiation dose for mutagenesis should consider these GR values to induce sufficient genetic variability while maintaining plant viability. Based on our findings, a dose around 60 Gy appears to be optimal for ‘Sabz’ fig cuttings.

### Multivariate analyses

**Fig 7:**
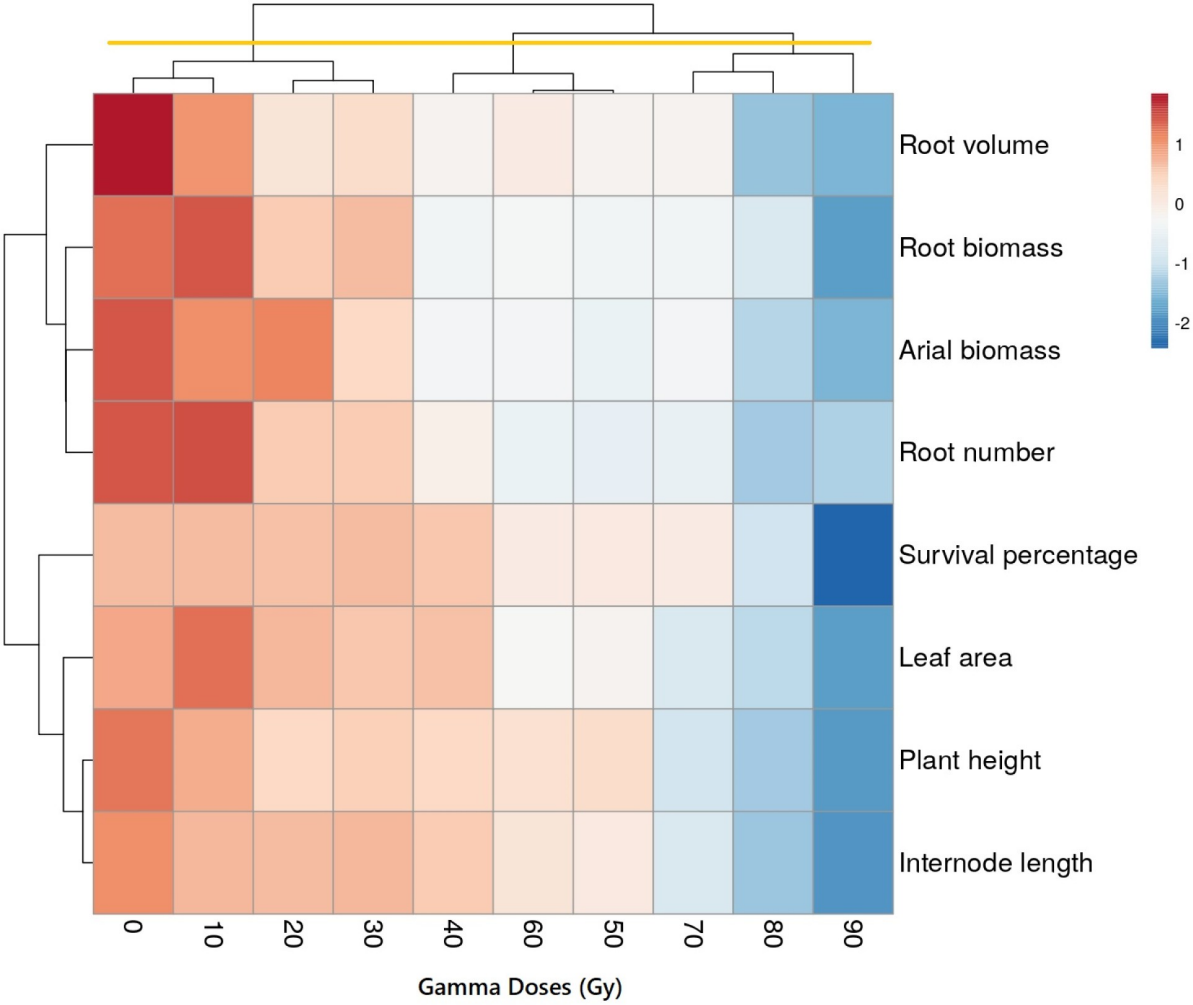
Cluster analysis of the studied traits in different gamma treatments in *Ficus carica* L. cv. Sabz. This approach showed that three distinct groups. The initial group encompassed the control treatment, as well as the 10, 20, and 30 Gy treatments. Notably, the control and low-dose treatments displayed a minimum negative effect in terms of leaf, shoot, and root characteristics compared to the other treatments. Furthermore, within this group, both the 10 Gy and control treatments outperformed the 20 Gy and 30 Gy treatments.

We utilized cluster analysis (Fig 7) to gain deeper insights into the behavioral patterns of various characteristics under a range of gamma-ray treatments.

The second group exhibited a complex structure, comprising two subgroups. The first subgroup featured the 50 and 60 Gy treatments. Notably, among the treatments within this subgroup, the 40 Gy treatment exhibited the highest value across all radiation treatments in this series. The third group, it included treatments with high doses of gamma radiation (70, 80, and 90 Gy). Surprisingly, despite this higher dose, the 70 Gy treatment demonstrated a suitable condition compared to the other treatments within this group. However, it’s worth noting that in terms of survival percentage, this particular treatment (70 Gy) was less effective than the 80 and 90 Gy treatments. Interestingly, when compared to other radiation treatments, the 90 Gy gamma irradiation exhibited pronounced strength.

**Fig 8:**
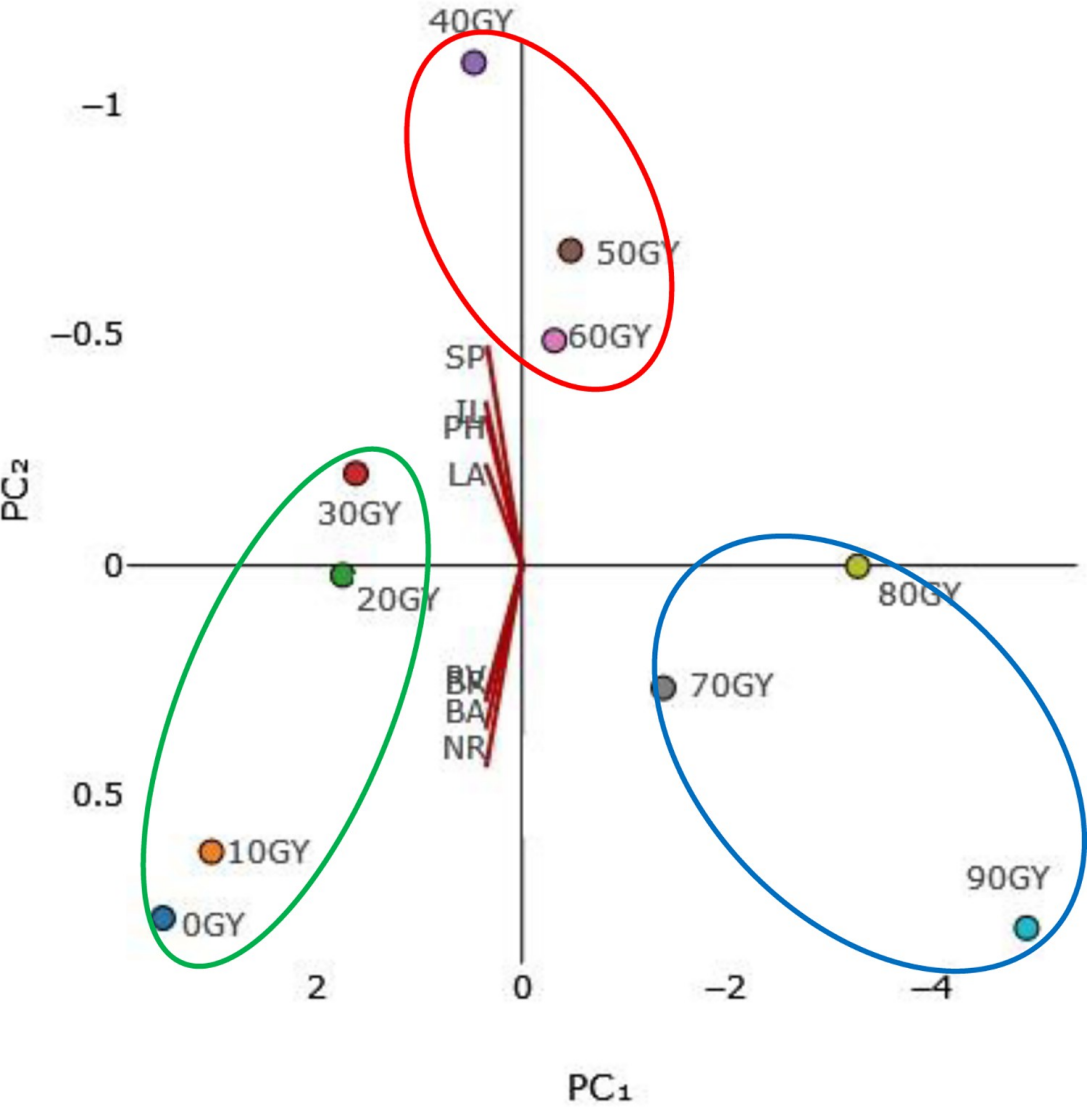
Principal component analysis (PCA) of the studied traits in different gamma treatments in *Ficus carica* L. cv. Sabz. (SP) Survival percentage, (PH) Plant height, (BR) Biomass of root, (NR) Number of root, (BA) biomass of aerial part, (RV) Root volume, (LA) Leaf area and (IL) Internode length.

Notably, the principal components analysis (PCA) results confirmed the findings of the cluster analysis (Fig 8).

Based on these results, three distinct groups emerged. Within the first group, we observed the inclusion of both the control treatment and low-dose treatments (10, 20, and 30 Gy). These treatments consistently displayed remarkably high values across most traits. The second group, corresponding to moderate doses (40, 50, and 60 Gy), the 40 Gy treatment stood out by attaining the maximum value for most of the traits examined. Lastly, the group consisting of high doses of gamma treatment (70, 80, and 90 Gy) revealed a most negative effect on fig plant. Surprisingly, the 90 Gy treatment exhibited a more adverse effect on vegetative traits compared to the other treatments in this high-dose category. Intriguingly, the 100 Gy treatment emerged as a source of vulnerability, representing fragile irradiation for the Sabz cultivar.

## Discussion

The determination of the LD₅₀ value is crucial in mutation breeding as it provides a benchmark for selecting appropriate radiation doses that balance mutation induction with plant viability. In our study, the LD₅₀ for ‘Sabz’ fig cuttings was calculated to be approximately 70 Gy, which is notably higher than the LD₅₀ values reported in some previous studies on figs and other plant species. For instance, Oualkadi and Taleb [21] reported an optimal radiation dose between 60 and 65 Gy for the ‘Achmrar’ fig variety, while Ferreira et al. [13] suggested a dose of 30 Gy for in vitro fig explants, indicating a lower LD₅₀ in their experiments. These differences in LD₅₀ values can be attributed to several physiological, biochemical, and molecular factors that influence the radiosensitivity of plants.

The variation in LD₅₀ values may stem from differences in the physiological status of the plant material used in different studies. Our research utilized one-year-old hardwood cuttings collected from mature ‘Sabz’ fig trees, whereas other studies may have used different cultivars, younger plant tissues, or in vitro cultures [7, 17]. The age and type of plant tissue significantly affect radiosensitivity; mature tissues often have more robust cell walls and developed protective mechanisms compared to juvenile or in vitro tissues, which can be more susceptible to radiation damage [22]. Moreover, the inherent growth habits and morphological characteristics of different cultivars can influence their response to gamma radiation [21]. The ‘Sabz’ cultivar may possess specific anatomical features, such as thicker cuticles or denser cell arrangements, that provide additional protection against radiation-induced damage [1]. Additionally, the physiological condition of the plants, including their nutrient status and water content at the time of irradiation, can affect their ability to withstand radiation stress [23]. Biochemical differences, particularly in antioxidant capacity and metabolic pathways, play a significant role in determining a plant’s tolerance to gamma radiation [24]. Gamma irradiation generates reactive oxygen species (ROS) within plant cells, leading to oxidative stress and cellular damage [25]. Plants with higher levels of antioxidant enzymes such as superoxide dismutase (SOD), catalase (CAT), and peroxidases can more effectively neutralize ROS, reducing the extent of oxidative damage [24]. The ‘Sabz’ fig may have a more robust antioxidant defense system compared to other cultivars, contributing to its higher LD₅₀ value. This enhanced biochemical capacity allows the plant to mitigate the detrimental effects of ROS generated by gamma radiation, maintaining cellular integrity and function [26]. Furthermore, the presence of secondary metabolites like phenolic compounds, flavonoids, and other antioxidants in figs can scavenge free radicals, providing additional protection [27].

At the molecular level, differences in DNA repair mechanisms and the activation of stress-responsive genes can significantly influence a plant’s radiosensitivity [28]. Gamma radiation causes various types of DNA damage, including single-strand breaks, double-strand breaks, and base modifications [24, 29]. The efficiency of DNA repair pathways, such as non-homologous end joining (NHEJ) and homologous recombination (HR), determines the cell’s ability to recover from such damage [30]. The ‘Sabz’ fig may possess more efficient or robust DNA repair mechanisms, enabling it to cope better with the genotoxic stress induced by higher radiation doses. Additionally, the activation of stress-related genes and signaling pathways can facilitate protective responses. For example, the upregulation of genes involved in cell cycle regulation, apoptosis inhibition, and DNA repair can enhance the plant’s resilience [31]. Epigenetic modifications, such as DNA methylation and histone modifications, also play a role in regulating gene expression in response to stress. Radiation-induced changes in the epigenome can lead to altered expression of genes involved in stress responses and developmental processes [32]. The epigenetic landscape of the ‘Sabz’ cultivar may contribute to its ability to withstand higher doses of gamma radiation.

Environmental conditions during and after irradiation, such as temperature, humidity, and light intensity, can influence the plant’s recovery and survival [24]. Our study maintained optimal greenhouse conditions post-irradiation, which may have facilitated better recovery and higher survival rates. In contrast, variations in environmental conditions in other studies could have contributed to lower LD₅₀ values [7, 21]. Differences in experimental methodologies, such as the dose rate of gamma radiation, the duration of exposure, and the type of radiation source, can also affect the results [17]. Acute vs. chronic exposure and the uniformity of radiation delivery are important considerations. Our study used acute gamma irradiation with precise dose measurements, which may differ from the protocols used in [17].

In our study, the GR₅₀ values for various growth traits of ‘Sabz’ fig cuttings ranged from 46 Gy to 67 Gy, indicating moderate sensitivity to gamma radiation. Specifically, the GR₅₀ values were 63 Gy for internode length, 67 Gy for leaf area, 56 Gy for plant height and aerial biomass, and between 46 Gy to 57 Gy for root traits such as root number, root volume, and root biomass. These values are crucial for determining the optimal radiation dose that induces desirable mutations while maintaining acceptable levels of growth reduction [20]. When comparing our GR₅₀ values with those reported in previous studies on figs and other plant species, notable differences and similarities emerge. Ferreira et al. [13] observed that in vitro fig plantlets exhibited a 50% reduction in both aerial component size and bud number at a much lower dose of 30 Gy, suggesting higher radiosensitivity in their study compared to ours. The discrepancy may be attributed to the use of *in vitro* plantlets, which are generally more sensitive to radiation due to their less developed protective structures and different metabolic states compared to ex vitro hardwood cuttings used in our research. Similarly, Oualkadi and Taleb [21] reported that the optimal radiation dose for the ‘Achmrar’ fig variety was between 60 and 65 Gy, aligning closely with our GR₅₀ values for ‘Sabz’ fig. This similarity indicates that different fig cultivars may exhibit comparable radiosensitivity when similar plant materials and experimental conditions are employed.

In studies involving other plant species, Ghasemi-Soloklui et al. [20] investigated the effects of gamma radiation on ‘Yaghouti’ grape and found GR₅₀ values for plant height, root length, and leaf area to be around 40 Gy. The lower GR₅₀ values in grapes compared to ‘Sabz’ fig suggest that grapevine may be more sensitive to gamma radiation. This increased sensitivity could result from differences in genomic composition, cell cycle regulation, and DNA repair mechanisms between the two species [24]. Conversely, Ulukapi and Ozmen [33] reported that common bean plants exhibited GR₅₀ values at doses higher than 100 Gy, indicating a higher tolerance to gamma radiation compared to ‘Sabz’ fig. Legumes like common beans may possess more efficient antioxidant systems and DNA repair mechanisms, enabling them to withstand higher radiation doses without significant growth reduction.

The variations in GR₅₀ values among different studies highlight several factors influencing plant radiosensitivity [20, 21, 24]: a) type of plant material: *In vitro* cultures are generally more sensitive to radiation than *ex vitro* plants due to their softer tissues, higher water content, and lack of protective barriers like cuticles and lignified cell walls [22]. This difference can lead to lower GR₅₀ values in studies using in vitro plantlets; b) genotypic differences: Genetic makeup plays a crucial role in determining radiosensitivity. Different species and even cultivars within the same species can exhibit varying levels of tolerance to radiation based on their inherent genetic traits affecting DNA repair capacity, antioxidant enzyme activities, and cell cycle control [34]. c) physiological state: The developmental stage and physiological condition of the plant material at the time of irradiation influence radiosensitivity. Actively dividing cells in meristematic tissues are more susceptible to radiation-induced damage [29]. Hardwood cuttings may have different sensitivities compared to seeds or seedlings due to variations in metabolic activity. d) environmental and experimental conditions: Factors such as dose rate, total exposure time, and post-irradiation environmental conditions (e.g., temperature, humidity) can affect the extent of radiation damage and subsequent recovery [31]. Consistency in experimental conditions is essential for accurate comparisons.

Our higher GR₅₀ values for ‘Sabz’ fig suggest that this cultivar has a moderate tolerance to gamma radiation, allowing for the use of higher doses in mutation breeding without causing excessive growth reduction. This tolerance is advantageous for inducing a broader spectrum of mutations, increasing the chances of obtaining desirable traits. Understanding these differences in GR₅₀ values is vital for optimizing gamma radiation doses in mutation breeding programs. It enables breeders to tailor radiation treatments according to the specific radiosensitivity of the plant material, ensuring a balance between mutation induction and plant viability. Our study contributes valuable information on the radiosensitivity of ‘Sabz’ fig, providing a foundation for future mutation breeding efforts in figs and offering comparative insights for other perennial fruit crops.

Our study observed significant changes in leaf, shoot, and root characteristics of ‘Sabz’ fig cuttings in response to increasing gamma radiation doses. These phenotypic alterations are indicative of the underlying physiological and biochemical disruptions caused by gamma radiation [35]. We observed that higher doses of gamma radiation (50–90 Gy) led to a significant reduction in leaf area, length, and width compared to lower doses and the control. This reduction in leaf size suggests that gamma radiation adversely affects leaf development and expansion. Gamma radiation induces oxidative stress by generating reactive oxygen species (ROS) such as superoxide radicals, hydrogen peroxide, and hydroxyl radicals within plant cells [22]. Excessive ROS can damage cellular components, including lipids, proteins, and nucleic acids, leading to impaired cell function and death [36]. In leaves, oxidative stress can disrupt chloroplast function and photosynthetic efficiency by damaging photosynthetic pigments and membranes [37]. This can result in reduced energy production for growth and development. Additionally, gamma radiation can interfere with cell division and elongation by causing DNA damage, leading to cell cycle arrest in the G_2_ phase [29]. Disruption in hormonal balances, particularly auxins and cytokinin’s that regulate leaf growth, may also occur due to radiation stress [38]. These factors collectively contribute to the reduced leaf size observed at higher radiation doses.

Plant height and internode length decreased progressively with increasing gamma radiation doses up to 60 Gy. A significant drop in shoot length was noted between 60 and 70 Gy, indicating heightened sensitivity at these doses. Gamma radiation-induced DNA damage in the shoot apical meristem impairs cell division and elongation, leading to stunted growth [31]. Radiation can alter the synthesis and transport of gibberellins and auxins, hormones essential for stem elongation and internode development [24]. Reduced hormone levels can inhibit cell elongation, resulting in shorter plants with decreased internode lengths. Moreover, radiation stress may activate stress-responsive genes and signaling pathways that prioritize survival over growth, leading to a shift in resource allocation away from elongation and development [23].

Root traits were particularly sensitive to higher radiation doses. Root number, length, volume, and biomass all decreased significantly with increasing gamma doses, with the most pronounced effects observed at doses above 40 Gy. However, low doses (10 Gy) appeared to enhance certain root characteristics, such as root length and fresh weight. Roots contain meristematic tissues with high rates of cell division, making them vulnerable to radiation-induced DNA damage [27]. This damage can result in cell cycle arrest, apoptosis, or mutations that impair root development [32]. Additionally, gamma radiation can disrupt the production of root growth hormones, such as auxins, and affect the expression of genes involved in root development. The observed enhancement of root traits at low radiation doses may be attributed to the hormesis effect, where low levels of a stressor stimulate beneficial physiological responses [39]. Low-dose radiation can activate antioxidant defenses and stress response pathways, promoting cell division and growth in roots [20, 24, 40]. However, as radiation doses increase beyond the hormetic threshold, the detrimental effects of oxidative stress and DNA damage outweigh any potential benefits, leading to impaired root growth.

The contrasting responses of root traits in ‘Sabz’ fig cuttings to different gamma radiation doses reflect a complex interplay of physiological, biochemical, and molecular mechanisms. At low doses (10 Gy), gamma radiation appeared to stimulate root growth, evidenced by improvements in root length and fresh weight. This stimulatory effect can be attributed to hormesis, where low levels of stress induce beneficial adaptive responses [41]. Low-dose radiation may activate stress response pathways and enhance antioxidant enzyme activities, such as superoxide dismutase and catalase, which mitigate oxidative stress by scavenging reactive oxygen species (ROS) [22, 27]. Additionally, there may be an increase in the synthesis or redistribution of growth-promoting hormones like auxins, crucial for root development and elongation [23]. The mild stress from low radiation levels could thus promote cell division and elongation in root meristems. In contrast, moderate to high doses of gamma radiation (40–90 Gy) resulted in significant reductions in root number, length, volume, and biomass. These inhibitory effects are likely due to extensive DNA damage in rapidly dividing root meristem cells, leading to cell cycle arrest, apoptosis, or mutations that impair cell division and elongation [29, 31]. High radiation doses generate excessive ROS, overwhelming the plant’s antioxidant defenses and causing oxidative damage to lipids, proteins, and nucleic acids, which disrupt cellular structures and functions essential for root growth [22, 23]. Hormonal imbalances may also occur, as gamma radiation can alter the synthesis, transport, and signaling of hormones involved in root development, such as auxins, cytokinin’s, and gibberellins, leading to inhibited root initiation and elongation [42, 43]. Moreover, radiation-induced changes in gene expression may upregulate stress-related genes while suppressing growth-promoting genes, further hindering root development [32, 35]. The differential sensitivity among root traits suggests that root initiation processes are more susceptible to radiation-induced disruptions than root elongation, possibly due to the complexity of initiating new root primordia compared to elongating existing roots. Additionally, under radiation stress, plants may reallocate resources away from root development to prioritize survival, limiting nutrient and carbohydrate allocation to roots and creating a feedback loop that further inhibits growth [25]. These findings highlight the importance of carefully selecting radiation doses in mutation breeding programs. While low doses may enhance root growth through hormesis, they may not induce sufficient genetic variability for effective breeding [10, 20]. Conversely, high doses may cause excessive damage, reducing plant viability and the success of subsequent breeding efforts [21, 44]. An optimal radiation dose should therefore balance the induction of beneficial mutations with the maintenance of root health and overall plant vigor. Understanding the contrasting responses of root traits to different gamma radiation doses provides valuable insights for optimizing mutation breeding strategies in figs and potentially other crops, enabling the development of new cultivars with desirable traits without compromising plant health and productivity [41, 45]. The utilization of cluster analysis and principal components analysis has shed light on the intricate relationships between the administered gamma-ray treatments and the observed characteristics. The study underscores the nuanced impact of radiation dosages on various traits and unveils compelling insights into the behavior of the studied plant material. This finding underscores how a higher dosage of radiation exerts a diminishing impact on the majority of the morphological features that were examined in this study. A method for reducing the number of data dimensions while minimizing information loss is principal component analysis (PCA). The process involves rotating the axes to increase their variance before converting the data into principal component values, also referred to as scores. In recent studies on grape [20] and stevia [19], cluster analysis and PCA methods were utilized to better understand how different plant parts responded to various gamma-ray treatments.

## Conclusion

The primary objectives of this study were to determine the optimal gamma radiation dose for inducing mutations in ‘Sabz’ fig cuttings and to assess the resulting phenotypic changes. By exposing fig cuttings to gamma radiation doses ranging from 10 to 100 Gy, we carefully monitored changes in growth patterns, leaf morphology, shoot traits, and root characteristics. Our key findings indicated that the lethal dose for 50% of the cuttings (LD₅₀) is approximately 70 Gy, with LD₂₅ and LD₇₅ calculated as 48 Gy and 95 Gy, respectively. Growth reduction (GR₅₀) values for critical traits such as plant height, leaf area, and root biomass ranged from 46 Gy to 67 Gy, demonstrating moderate sensitivity to gamma radiation. Root traits were more sensitive to radiation compared to aerial parts. Based on the LD and GR analyses, we identified 60 Gy as the optimal gamma radiation dose for inducing mutations in ‘Sabz’ fig cuttings while maintaining acceptable levels of survival and growth. These findings directly address our objectives and provide valuable insights for fig breeding programs aiming to develop new cultivars with improved traits through mutation breeding.
